# Precision medicine: The use of tailored therapy in primary immunodeficiencies

**DOI:** 10.3389/fimmu.2022.1029560

**Published:** 2022-12-08

**Authors:** Marta Valente Pinto, João Farela Neves

**Affiliations:** ^1^ Primary Immunodeficiencies Unit, Hospital Dona Estefânia, CHULC-EPE, Lisbon, Portugal; ^2^ Centro de Investigação Egas Moniz (CiiEM), Instituto Universitário Egas Moniz (IUEM), Quinta da Granja, Monte da Caparica, Caparica, Portugal; ^3^ CHRC, Comprehensive Health Research Centre, Nova Medical School, Lisbon, Portugal

**Keywords:** precision medicine, primary immunodeficiencies, NGS, PIRD, target therapies

## Abstract

Primary immunodeficiencies (PID) are rare, complex diseases that can be characterised by a spectrum of phenotypes, from increased susceptibility to infections to autoimmunity, allergy, auto-inflammatory diseases and predisposition to malignancy. With the introduction of genetic testing in these patients and wider use of next-Generation sequencing techniques, a higher number of pathogenic genetic variants and conditions have been identified, allowing the development of new, targeted treatments in PID. The concept of precision medicine, that aims to tailor the medical interventions to each patient, allows to perform more precise diagnosis and more importantly the use of treatments directed to a specific defect, with the objective to cure or achieve long-term remission, minimising the number and type of side effects. This approach takes particular importance in PID, considering the nature of causative defects, disease severity, short- and long-term complications of disease but also of the available treatments, with impact in life-expectancy and quality of life. In this review we revisit how this approach can or is already being implemented in PID and provide a summary of the most relevant treatments applied to specific diseases.

## Introduction

The concept of precision medicine means, in simple words, the use of a tailored medical approach not only based on the disease signs and symptoms, basic laboratory workup and imaging, common to the majority of the patients, but also considering the lifestyle, biological and genetic characteristics of the individuals that we are targeting (1).

The definition of this concept can differ between peers, but the final aims are to provide better and more precise diagnosis, by using biomarkers that can help to predict disease phenotype and behaviour (2) as well as to plan more efficacious and targeted treatments to each patient, by anticipating the response to intervention (1, 2).

The methods and the number of conditions used as part of a precision medicine approach increased and currently is considered how future medicine should be implemented, not only by the scientific community but also with strong political involvement (1).

The use of this approach is particularly important in rare and severe diseases like primary immunodeficiencies (PID), a group of heterogenous disorders caused by monogenic defects leading to disfunction of the immune system, that can translate to loss of the ability to promote innate and/or adaptative immune responses with increased susceptibility to infection, auto-immunity, allergy, auto-inflammatory diseases and predisposition to malignancy (3, 4).

The discovery of the first PID was attributed to Ogden Bruton in 1952, when he described a condition in a child with recurrent life-threating infections likely associated to the complete absence of gamma globulin, with no identified aetiology (5). This condition, named agammaglobulinemia, was not only one of the first full description of a disease understood as PID but also the first step for a precision medicine like-approach. Bruton et al. used successfully subcutaneous gamma globulin as a replacement treatment, monthly, preventing further severe infections (5). This patient also contributed for a wider knowledge, nowadays a current practice, that treatments might need to be adjusted, with the progressive adaptation of the gamma-globulin doses as the patient weight increased (6).

**Figure 1:**
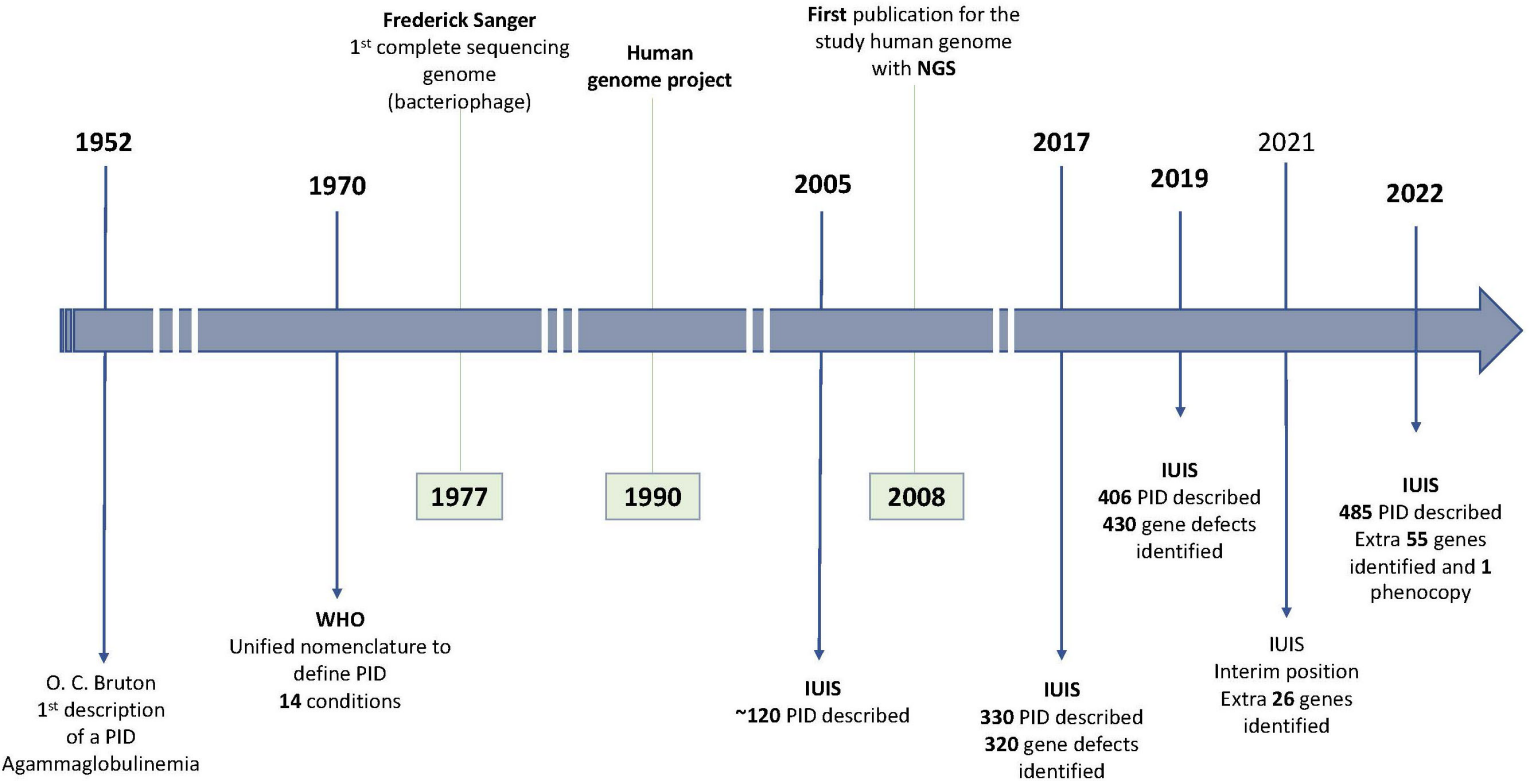
Primary Immunodeficiencies (PID) discovery chronology and gene sequencing landmarks IUIS, International Union of Immunological Societies; NGS, Next generation sequencing; WHO, World Health Organization.

After this first description, an increased number of diseases affecting the innate and adaptative immune response were described. In 1970 the WHO provided the first classification for primary immune disorders including a total of 14 conditions (7). Throughout the years, with the advent of novel methods like next generation sequencing (NGS), the number of identified causative effects increased exponentally. After more than three decades the total number of recognized PID were more than 120, arranged in eight categories, according to the 2005 report of the International Union of Immunological Societies (IUIS) (8). In IUIS 2022 classification, a total of 485 human inborn errors of immunity, adding 55 novel monogenic defects and one auto-immune phenocopy were described (9), representing an increase of almost 150 conditions since the update in 2013 where just a little over 330 diseases were acknowledged in the classification and an increase of around 80 conditions since the 2019 classification. **Figure 1** illustrates the evolution in the discovery of new PID throughout the years.

Increased susceptibility to infection is a phenotype well recognised in PID, but in recent years the identification that some inborn errors of metabolism can present with autoimmunity and autoinflammation events changed the therapeutic approach to these patients. The primary immunoregulatory disorders (PIRD) represent the group of PID conditions with a predominant phenotype of autoimmunity and autoinflammation, opening the path for a more generalised use of immunoregulators (10). It is fundamental that three of the main phenotypes in PID, with a likely but variable overlap, can be included in the therapeutic approach to these patients.

Identification of causative mutations and the understanding of the underlying affected immune pathways allows the development of treatments directed to the correction of the mutation or to the defective immune mechanism, moving forward to a more generalised implementation of precision medicine in PID.

**Figure 2:**
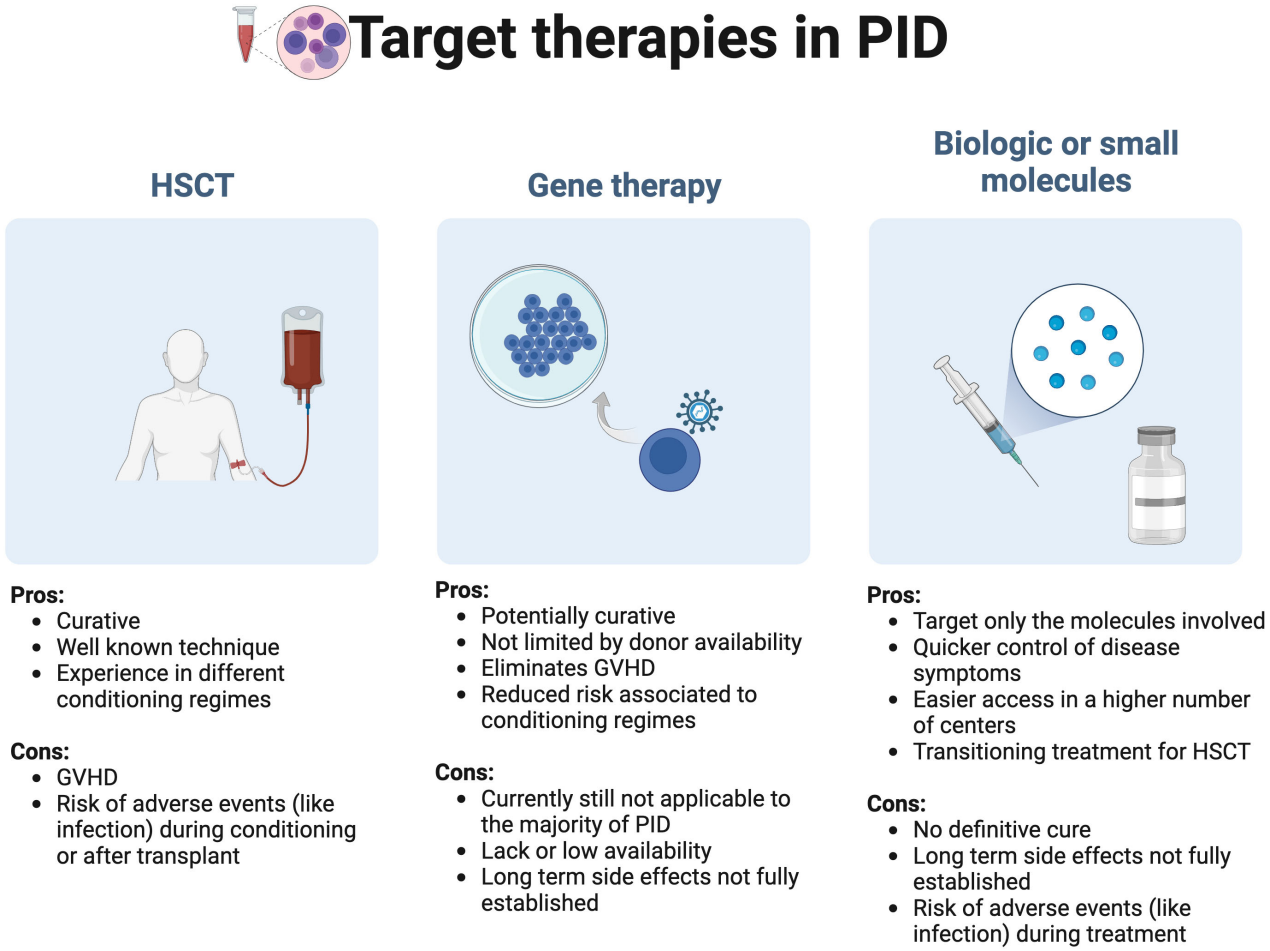
Target primary immunodeficiencies (PID) therapies in precision medicine: A general overview GVHD, Graft versus host disease; HSCT, hematopoietic stem cell transplant.

When exploring precision medicine targeted therapies in the field of PID, at least three main fields need to be considered: HSCT, gene therapy and the use of biologic or molecules targeting a specific immune pathway/cell function (**Figure 2**).

As a broader therapy of PID, it is debatable if HSCT should be considered a precision therapy, but like other publications, we have included it in this review considering that historically it was the first therapeutic approach with the aim to correct inborn errors of immunity. HSCT is still considered the first line curative treatment in PID, since data in gene therapy, a method that can correct the gene defect, is still limited (11). A full guideline on the use of HSCT in PID was recently published by the EBMT/ESID inborn errors working party (11).

Gene therapy consists in using patients’ blood stem cells that carry a specific mutation and correct that mutation by using a retroviral vector, simple or complex, that delivers the non-pathogenic gene to integrate the host cell genome. This process allows a correct transduction and a long expression of the delivered gene variant (12). After retroviral treatment the cells are infused again into the patient carrying the corrected gene (12, 13). The use of gene therapy is considered a valid alternative if no human leukocyte antigen (HLA) related donor is available, with the advantage of eliminating the graft versus host event and reduce the risks associated to the conditioning regimes used in allogenic HSCT (12, 14, 15). Several biologic drugs and small molecules are currently available or under evaluation and can be used as a single therapeutic option, as a complement to other treatments or a bridge for HSCT.

In this review we will focus first on describing a group of PID conditions and the specific targeted treatments that they benefit from, and end with a general overview of the main therapeutic precision medicine treatments available in the field of PID.

## Overcoming susceptibility to infection with precision medicine

One of the first phenotypic characteristic that is recognized as being associated to PID patients is the increased susceptibility to infections, associated to defects in the innate, humoral and/or cellular immunity. Several innate immunity components, including phagocytes, complement, inflammation related proteins and natural killer (NK) cells can be affected in PID. The mutations in cells or other molecules of this first line of defence are associated to a higher susceptibility to a wider range of infectious agents, including the risk of invasive bacterial infections, fungal infections, mycobacterial disease and viral infections (4, 16, 17). Phagocytes are able to recognize antigens, more specifically pathogen associated molecular patterns (PAMPS) through cell surface receptors or antimicrobial peptides (AMP), that work as soluble receptors encoded by intact germline genes (16, 17).

T and B lymphocytes are the main players in adaptative immunity and are involved in antibody/humoral and cellular responses that allow the elimination of the pathogen and development of immunological memory (16). In general, it is recognised that individuals with antibody deficiencies have an increased susceptibility to local and invasive infections caused by capsulated bacteria and the defects in T cell immunity are associated to an increased susceptibility to viral and fungal infections.

Prophylactic therapy with antibiotics, antifungal, antiviral and antiparasitic drugs are wider implemented. These include a wide range of agents and sometimes the same drug is used not only for prophylaxis but also for treatment of a confirmed or suspected infection (18).

### Immunodeficiencies affecting cellular and humoral immunity

Immunodeficiencies that affect humoral and cellular immunity are associated with an increased risk of severe, life-threatening infections. Severe combined immunodeficiency (SCID) is the most severe form of these conditions and is immunophenotypically characterized primarily by a CD3 T cell profound lymphopenia (<300 cells/microliter or less that 20% of CD3+ T cells), that can be accompanied by B and NK cell defects (4). Early identification of this condition, before any infectious life-threatening complications can be fundamental for successful implementation of curative treatment and survival (19). T-cell receptor excision circles (TREC) are the result of T-cell receptor gene rearrangement in the thymus formed during the development of new T cell lymphocytes. The absence of TREC could be indicative of low levels of T-lymphocytes from the self, with high sensitivity for detection of SCID, a neonatal screening tool currently used in several countries, allowing an early diagnosis of SCID (19, 20). Allogenic hematopoietic stem-cell transplant (HSCT) was recognised as the first choice as SCID curative treatment (11, 21), although currently other alternatives are available and designed to target a specific genetic defect, like gene therapy. Adenosine deaminase (ADA) deficient SCID is the classical example of the use of other tailored therapies, like enzyme replacement treatment with a polyethylene glycoconjugate adenosine deaminase (22) and autologous HSCT with gene correction in hematopoietic stem cells (14). In PID, this treatment was first used in SCID patients with adenosine deaminase deficiency (ADA), in the early 1990’s (23), using a gamma-retrovirus (24). This approach seemed to correct the deficiencies in the immune system that were associated to severe and fatal infections but did not correct other phenotypic features affecting nervous system (25). With the generalization of the use of this type of vector in other PID research studies, it was identified a higher risk of monoclonal expansion, with activation of genes associated with malignancy processes (26, 27). This was attributed to the gamma-retrovirus vectors being integrated nearby transcriptional start sites, allowing the viral long terminal repeat enhancer to change the expression of proto-oncogenes, increasing genotoxicity (28). To overcome this finding the use of the complex retrovirus, like lentivirus, was considered, since they have a better ability to regulate the process of transcription, improving safety (12). One of the main explanations for the lower toxicity of the lentivirus was the absence of an active viral long terminal repeats, the integration of the viral vector at different site when compared with retroviral vectors, and the lack of ability to activate proto-oncogenes (29).

In the specific case of ADA-SCID patients, this genotoxicity was not identified with gamma-retroviral vector and this treatment was approved by EMA in 2016 (14). Despite this fact, it was reported that a patient with ADA SCID developed a lymphoproliferative condition 20 years after gene therapy with a simple gamma-retrovirus (25). In a review by Ferrari et al. from 2020 more than 30 gene therapy clinical trials on monogenic diseases (phase I to phase III) were registered, including X -linked SCID (X1-SCID) (*ILR2G*), ADA-SCID (*ADA*), Wiskott- Aldrich (*WAS*), X-CGD (*CYBB*), Leukocyte adhesion deficiency (*CD18*) and Artemis-deficiency (ART) SCID (*DCLRE1C*) (30).

The first studies in X1-SCID, that use a gamma-retrovirus vector, showed an adequate T cell response (31). Findings of insertional oncogenesis, after integration of the vector in a proto-oncogene domain causing an increased expression of these domains (28), prompted the research of lentivirus as vectors in X1-SCID patients (30). This vector was used in a trial including eight paediatric patients after being conditioning with low doses of busulfan (32). In seven of those infants, it was described a reconstitution of B and T lymphocytes, allowing to stop gammaglobulin infusions (32). The quicker T-cell reconstitution that was reported in the study in comparison with HSCT (31) was hypothesised as predictive marker of survival and long term T cell reconstitution (15).

Gene therapy for ART-SCID patients is also under investigation. A pre-clinical study in a mouse model using a self-inactivating lentivirus vector that expresses human DCLRE1C cDNA, designated *AProArt*, showed a reconstitution of T and B cell lymphocytes, with no insertion at proto-oncogenic sites (33). Using the same vector, and a low dose busulfan as a conditioning treatment, an ongoing clinical trial (ClinicalTrials.gov Identifier: NCT03538899) in a paediatric population showed in the preliminary evaluation a reconstitution of T cell population, with no insertional oncogenesis (25).

SCID caused by mutations in the recombinant activating genes RAG-1 and 2 have been also evaluated as candidates for gene therapy. This approach was not successful initially, with two main problems identified: 1) a high number of insertional mutagenesis when using a retroviral vector, 2) a low expression of the non-mutate RAG-1 gene, when using a self-inactivating lentivirus, not allowing a correction of the SCID, leading to a “leaky” SCID/Omenn-like phenotype (34). A pre-clinical study showed the possibility of using a platform that optimized the codon of RAG1 gene, leading to a higher expression of RAG-1 corrected gene (34). Currently a non-randomized, phase I/II clinical trial is ongoing (ClinicalTrials.gov Identifier: NCT04797260) with the aim to infuse CD34+ cells treated with lentivirus vector targeting RAG-1 gene.

### Congenital defects of the phagocytes

Several conditions affect phagocytes, both in number and/or function (4). Congenital neutropenia includes a group of syndromic and non-syndromic conditions with more than two dozen gene defects already described (4, 35, 36). In terms of patient management, prophylactic therapeutics to prevent viral, fungal and bacterial infections are widely used (35) and it is generally recommended the administration of granulocyte colony stimulating factor (G-CSF) to first increase and then to maintain the neutrophil levels. This last treatment contributes to an improvement of life expectancy but is associated to an increased risk of myelodysplastic syndrome and acute myeloid leukaemia (35, 37). Although the use of hematopoietic growth factors can only be considered a semi-tailored treatment in patients with congenital neutropenia, it has been understood in recent years that the response differs between patients and for specific mutations, allowing to tailoring the doses or even the type of myeloid hematopoietic growth factors (36, 37). The analysis of severe chronic neutropenia international registry (SCNIR) identified a group of mutated genes, *ELANE, JAGN1, SRP54*, associated to a non-response (absolute neutrophil count (ANC) below 500/μL) or to a partial response (ANC 500-999/μL) to G-CSF doses of 20 mg/kg/day or higher (37), showing that the use of G-CSF seems not to be effective to all type of genotypes. Homozygous mutations in *CSF3R* (*R547X CSF3R*), affecting the extracellular domain of G-CSF receptor, were refractory to this treatment and only responding to granulocyte-macrophage colony stimulating factor (GM-CSF) (36, 37). These two hematopoietic growth factors differ not only in structure, but also in the biologic effect, with GM-CSF receptors being expressed in a higher number of cells, not only neutrophils and monocytes, but also in eosinophils, dendritic cells and basophils (38).

Leukocyte adhesion deficiency (LAD) type-1 is a rare condition cause by a mutation in the *ITGB2 gene*, that is associated to a decreased expression of CD18 (39). Several trials for targeted treatments are ongoing, considering that after HSCT these patients can still develop autoimmune manifestations target anti-inflammatory drugs can have a major role (39).

Allogenic HSCT has been considered for conditions with specific lesions, like *ELANE* and *HAX1* mutations that are not responsive to treatment with these hematopoietic growth factors and with higher risk to develop a malignancy (40). Gene therapy studies have focused on the correction of autosomal dominant mutations in the *ELANE* gene. A study targeting *ELANE gene exon 4*, using CRISPR-Cas9 ribonucleoproteins and adeno-associated virus in haematopoietic stem and progenitor cells of a paediatric patient with a heterozygous missense c.515T>T mutation (L172P), showed a restoration of the neutrophil differentiation when transplanted in a mice model, opening the possibility to use gene therapy as a tailored curative alternative treatment (41).

Chronic granulomatous disease (CGD) is also included under the congenital defects of the phagocyte (4) caused by a defect in the sub-components of nicotinamide adenine dinucleotide phosphate (NADPH) oxidase complex, affecting the production of reactive oxygen species by those cells. In terms of genetic causes it can be X-linked, affecting the main membrane subunit oxidase gp91phox protein, encoded by *CYBB* gene, or autosomal recessive (AR) with defect on p47phox (*NCF1* gene) and p67phox (*NCF2* gene) cytosolic subunits or in the membrane protein p22phox (*CYBA* gene) (42–44). In 1960’s Holmes et al. identified a defect in the phagocytes function (45) associated to an impaired ability to promote intracellular bacterial killing (46). It was also described a possible higher susceptibility to a narrow group of specific microorganism like *Staphylococcus* (45), a first step to direct treatment and in the future a prophylactic approach to infection.

The identification of elements, like cytokines that are part of the bacterial killing pathway was used to identify potential therapeutic targets. Gamma-interferon (*IFN-γ*) is a macrophage-activating cytokine, produced by T lymphocytes and natural killer (NK) cells (47). In the first clinical trial using a recombinant human gamma-interferon (*rIFN-γ*), a clear association was found with the reduction of the number of severe infection episodes, hospitalisation, and mortality and the use of this treatment (48, 49). Although some studies failed to prove the restorative effect in the oxidative metabolism of the phagocytes when using rIFN-γ, others have shown an increase in superoxide and an increase in the production of nitric oxide by polymorphonuclear neutrophils, a compound with cytotoxic and bactericidal activity (50, 51). In a study by Condino-Neto et al, in patients with X-linked CYBB splice mutations, a phenotypic presentation typically less severe, the use of rIFN-γ lead to an increase in the ratio of spliced transcripts, causing a higher number of spliced mRNA with a normal translation of the gp91-phox protein (52). Those findings contributed to the recommendation for the use of rIFN-γ specifically in patients with CGD due to X-linked splice mutations and not as a routine or universal treatment (44).

HSCT is the standard current available curative treatment for CGD patients, like many other PID, but as in other diseases the intensive conditioning regime is associated to a higher number of severe infections and the less intensive regimes to a higher number of episodes of graft versus host disease (GVHD) (11, 53). Although the risks associated to HSCT, it was reported in total of 386 CGD patients that underwent HSCT a median overall survival rate of 91% (53). Another study that evaluated 104 patients, 50 of whom received a HSCT, showed an 88% survival rate after 2.2 years. Considering that 12% of the patients died after a severe infection, younger individuals and patients without any previous complications were reported to have the best survival rates in this group (54).

Still under experimental phase, gene therapy is being investigated as a potential treatment in CGD patients (11). Most recent preliminary data using a lentiviral gene therapy in patients with severe X-linked CGD showed that the patients restored NADPH-oxidase activity, with no associated genotoxicity and in the three years follow up no severe infections were reported (55). Also, phase I/II gene therapy clinical trials in p47phox-CGD patients are in setup (ClinicalTrials.gov Identifier: NCT05207657) using a pCHIM-p47 (lentiviral vector transduced CD34+ cells). A previous pre-clinical trial that developed a lentiviral vector with a codon optimised of the human NCF1 gene showed an effective gene expression of p47phox, translated in a restore NADPH oxidase function and consequent reduction of bacterial load in challenged mice (56).

### Defects of the intrinsic and innate immunity

An increasing number of genes have been identified as cause of the defects in intrinsic and innate immunity. IFN-γ is a fundamental cytokine that acts against intracellular pathogens and in terms of innate immune response is produced by NK and NK T cells. The JAK-STAT pathway mediates the IFN-γ induced signaling, with STATs working as signal transducers and activators of transcription (57, 58). STAT1 inborn errors are a good example in how the inheritance pattern and the impact of the defect in the protein functionality (meaning a loss of function (LOF) or the gain of function (GOF) of this transcription factor), can lead to different disease phenotypes (4, 59). STAT1 is activated by IFN-γ after a series of tyrosine phosphorylation events are initiated allowing a translocation of STAT1 homodimers to the nucleus to activate the effector genes (59). Autosomal recessive defects can lead to a complete or partial defect STAT1 function and increase the susceptibility to intramacrophagic bacteria, including *Mycobacteria* and *Salmonella*, but can also be associated with increased susceptibility to viral infections, particularly herpes virus (59). The autosomal dominant mutations with a STAT1 LOF are responsible for the Mendelian susceptibility to mycobacterial disease.

Autosomal dominant mutations, typically missense (60), can cause GOF of these transcription factor, causing more commonly chronic mucocutaneous candidiasis, typically caused by *Candida albicans*. The increased levels of IFN-γ are also associated to the increased risk of autoimmunity, present in more than 50% of the patients (59, 60).

As previously described for other PID, prophylactic treatment against the most common infections, in this case anti-fungal fluconazole, is common practice (60). In STAT1 defects, HSCT have been attempted as a curative treatment, but a high number of complications with fatal outcomes particularly in STAT1 GOF mutations, probably due to patients’ pre-transplant physical conditions, confirmed the need for the use of other methods (60). The current approach in the pathway of precision medicine is the use of JAK1/2-kinase inhibitors in patients with STAT1 GOF mutation (61). JAK1/2-kinase inhibitor Ruxolitinib improved severe mucocutaneous candidiasis episodes, but only during active treatment, implying the need to be used long-term (61). Although more data are needed regarding the long-term use and generalise benefit of this treatment (62), in responsive patients, this treatment seems to improve the pre-transplant condition working as a bridge to HSCT, potentially improving the final outcome (61). Currently, several JAK inhibitors are approved or under development targeting different conditions, with new generation JAK inhibitors targeting only one JAK, conferring higher specificity and probably less adverse effects (58). A general overview of JAK inhibitors is described in the sections below (**Target therapies in precision medicine).**

The WHIM syndrome is characterized by the presence of warts, hypogammaglobulinemia, infection and myelokathexis that leads to neutropenia due to the retention of these cells’ precursors in the bone marrow (63, 64). WHIM is the result of an autosomal dominant GOF mutation in *CXCR4*, a chemokine receptor gene (65, 66). The chemokine CXCL12 expressed in leukocytes binds exclusively to CXCR4 and the mutation contributes to the lack of down-regulation of CXCR4, increasing the signaling of this receptor (64, 67). It is the lack of balance between the oversignaling of CXCR4, expressed in the blood cells, and the CXRC2 in the bone marrow, two receptors responsible for the migration of neutrophils between the two compartments, that leads to the myelokathesis (67).

The most common therapeutics used in this condition are replacement treatments particularly G-CSF and intravenous gammaglobulin, but these treatments do not prevent long term complications of the disease, like squamous cell carcinoma as a consequence of Human papillomavirus (HPV) infection (64). With the understanding of the pathophysiology behind this syndrome, targeted treatments with a competitive antagonist of CXCR4 (Plerixafor) have been explored (66, 67). A decrease in the number of infective episodes was reported, with a complete or partial resolution of benign and malignant lesions caused by HPV and better quality of life (66). Mevorixafor is also a selective antagonist of CXCR4 receptor, that contributes to the trafficking of white blood cells from the bone marrow to peripheral blood (68). This drug in a dosage of 400 mg a day in an adult population showed a good safety profile, increased the absolute neutrophil count (ANC) and contributed for a long-term reduction of the number of cutaneous warts and infection rates (68). The oral formulation of Mevorixafor is the main advantage of this treatment, particularly relevant in the paediatric population (68).

## Precision medicine in PIRDs

Autoinflammatory and autoimmune events are part of specific PID conditions, included in PIRD phenotype. It was proposed that conditions grouped as PIRD could be grouped by disease phenotype (10): Tregopathies, autoinflammatory syndrome, hyperinflammation disorders with predisposition to hemophagocytic lymphohistiocytosis (HLH), debris defects, non-malignant lymphoproliferation, hematopoietic malignancies, congenital atopic hypersensitivity, IBD and rheumatologic diseases.

Some of the conditions included in the PIRDS phenotype are explored below.

### Very-early onset inflammatory bowel disease

Inflammatory bowel disease (IBD) is a condition characterized by a chronic inflammation of the gastrointestinal tract, that is classically divided in Crohn’s disease (CD) and ulcerative colitis (UC), due to distinct histological, immune and clinical manifestations of each disease (69).

In patients with very early onset IBD (VEO-IBD), it is challenging to determine the type of IBD, with a high number of patients classified as IBD-unclassified (70).

In 2009, Glocker et al. described for the first time the presence of LOF mutations in *IL10RA* and *IL10RB* genes in children with very early onset colitis (71). Considering that IL-10 inhibits the production of pro-inflammatory cytokines, in the case of VEO IBD, it was hypothesised that the LOF mutations in the *IL10R* gene could lead to impaired signaling by IL-10, causing an inflammatory process in the intestinal immune system (71).

Since the discovery that mutations in the *IL10RA* and *IL10RB* genes are a monogenic cause of VEO-IBD (71), more than 60 causative genes of IBD phenotype were identified, with the defects affecting specific components of the immune system including the epithelial defence barrier (like *TTC7A, IKBKG, ADAM17* genes), T and B cells and other complex function (like *LRBA, CTLA4, IL-21, WAS* gene), phagocytes and NADPH oxidase complex (like *CYBB, G6PC3, SLC37A4* genes), other genes involved in loss of tolerance, causing auto-inflammation (like *XIAP, HPS1, MEFV, TNFAIP3* genes) and immune dysregulation (like *FOXP3, IL10RA, IL10RB* genes) (69, 70, 72).

The increased number of monogenic mutations strengthen the idea that the use of a tailored therapeutic and not conventional IBD treatment is an adequate approach. This consideration is also valid to monogenic diseases that present with an IBD-like phenotype. With the identification of IL10R mutation as monogenic cause for IBD, HSCT was used successfully as a curative treatment in around 80% of patients (71, 72). This treatment is also considered for other defects with an IBD phenotype, including severe X-linked CGD (around 96% successful) and XIAP deficiency (around 82% successful) (70, 72). HCST seems not to be useful for all gene defects and in the case of *TTC7A* defect no improvement in intestinal manifestations was reported after transplant, independently of immune reconstitution (72, 73). Similar to this, HSCT does not ameliorate IBD despite correcting the immune defect in patients with IKBKG deficiency, suggesting that it is the epithelial deficiency of NEMO, and not the hematopoietic cells deficiency that leads to intestinal inflammation (74)

The use of IL-1 inhibitors can also be considered in individuals with IL-10 mutations (75). The rational for the use of this drug is the assumption that the IL10R LOF mutations can interfere with the inflammasome activation, causing an increased production of the pro-inflammatory cytokine IL-1 (71).

Targeted treatments have also been explored in VEO-IBD and immunomodulator drugs are now considered for specific gene defects, including: *CTLA4/LRBA, MVK/NLRC4* and *HPS1/6* (72).

### Common variable immunodeficiency

The common variable immunodeficiency (CVID) is a heterogenous group of diseases that has been associated with specific genetic defects, that can present with low levels of IgG and IgA with or without low IgM levels, absent or lower antibody responses to vaccination and a low switched memory B cells in individuals older than 4 years of age (76). It was the implementation of more advanced genetic sequencing methods that allowed the discovery of specific mutations previously classified as CVID, a fundamental step to treatment tailoring.

Abolhassani H et al. used of sequencing to identify novel mutations in a total of 591 patients from three different countries and continents, with a previous diagnosis of CVID according to ESID criteria (77). From this population, between 31 to 54% of the patients had at least one mutation identified considered to be pathogenic, targeting different pathways and defence mechanisms of B-cell populations (77). The most common variants had some differences across countries, with mutations in the transmembrane activator and CAML interactor (*TACI*) gene considered to be predominant in the USA patients, mutations in the *LBRA* gene and heterozygous variations in the *TACI* gene in the Swedish cohort and biallelic mutations in the *LBRA* gene in the Iranian population (77). Lipopolysaccharide responsive beige-like anchor (LRBA) mutations are associated to lower levels of cytotoxic T lymphocyte-associated protein 4 (CTLA4), due to the increased turnover of this protein (78). The identification of these mutations allows the use of specific biologic treatments targeting a pathway or cell function, described below, and allows a better understanding of the indications and timing to propose HSCT as a curative treatment (11).

### Activated PI3 kinase delta syndrome

Activated PI3 kinase delta syndrome (APDS) is a condition characterised by immunodeficiency with senescent T cells and recurrent infections (more commonly bacterial infections affecting the respiratory tract and infections from the Herpes family), lymphoproliferation with hepatosplenomegaly, autoimmunity events, malignancy like lymphoma and neurological involvement (79, 80). This condition results of GOF mutations in the gene that encode phosphoinositide-3-kinase δ (PI3Kδ) and depending on the subunit targeted can be classified as APDS1 (catalytic subunit p110δ) or APDS2 (regulatory subunit p85α) (79–81). This protein is a cellular messenger that binds to multiple receptors of immune cells from the adaptive and innate immune system and regulates different cell functions, activates cytokine stimulation receptors and cytokine production, cell survival and metabolism (82, 83).

As previously described for other PID, HSCT has been the option to treat successfully APDS patients (80). Although not fully targeting the defect, treatments directed to the disease outcomes like lymphoproliferative events have been used, including anti-monoclonal CD20 antibody rituximab and the macrolide compound that inhibits the activation of mTOR, sirolimus (80). With the generalised use of NGS and identification of the mutations that are associated to the disease specific mechanism, selective PI3 kinase delta inhibitors were developed. Leniolisib is a small molecule, given orally, that inhibits the p110δ subunit of PI3K, leading to the inhibition of 3’4’5’trisphosphate (PIP3) (81, 83). Previous trials have shown a regression of the lymphoproliferation events, with reduction of lymph nodes and spleen and with an improvement of the immunological markers (83). Another PI3 kinase delta inhibitor being studied is nemiralisib, an inhaled formulation, particularly targeting the respiratory manifestations (80, 81).

### STAT3 GOF mutations

Similar to what was previously described for STAT1 defects, STAT3 LOF or GOF mutations have been identified and are associated to immune dysregulation (58, 84). This protein is activated as a transcription factor, after IL-6 binds to this receptor inducing phosphorylation of JAK kinase, resulting in the phosphorylation of STAT3 and the dimers migrating to the nucleus (85). After activation, this transcription factor regulates intracellular signaling of different cytokines and growth factors, indirectly activates Th17 responses and has a role in regulating T cells (86).

GOF mutations in STAT3 influence other molecules of this pathway, including the lack of inhibition of suppressor of cytokine signaling 3 (SOCS3) that not only regulates STAT3 activity, but also STAT1 and STAT5 (87).

The role of STAT3 in different immune pathways explains the variation of disease manifestations between patients, that can overlap with other PID conditions (88). The disease manifestations include autoimmunity, lymphoproliferation, hypogammaglobulinemia and increased risk of infectious diseases (85). Lymphoproliferation, characterized more commonly by hepatosplenomegaly and less frequently by malignancy, is one of the most common described features of STAT3 GOF mutations and seems to result of the interference in the transcription of genes that contribute for the regulation of cell proliferation and apoptosis (89–91).

As described for STAT1, JAK inhibitors can reduce the GOF effects of STAT3, although all interference at the top of the pathway can inhibit other relevant signaling, with higher number of side effects, including higher susceptibility to infection (58).

JAK inhibitors, including ruxolitinib and tofacitinib, were used with success in patients with STAT3 GOF mutations to control autoimmunity/immune dysregulation events (92–94). An ongoing clinical trial (NCT04774224) is evaluating the effect of baricitinib (JAK-1 and 2 inhibitor) in new onset type 1 diabetes (T1D). The investigators hypothesise that this treatment can delay disease progression by preventing the death of insulin-producing beta cells. The destruction of these cells occurs by an auto-immune mediated mechanism with involvement of the JAK-STAT signalling pathway. In the mouse model the use of JAK-1 and 2 inhibitors were able not only to prevent the development of T1D and even promote cure T1D mice (95).

Tocilizumab, a monoclonal antibody against IL6 receptors, also interferes in the JAK-STAT pathway and is frequently used in combination with JAK inhibitors, with good response in patients with lung disease and enteropathy (85, 92).

In the field of precision medicine, development of STAT inhibitors can minimize the events associated to the inhibition of JAK, higher in the pathway regulation. STAT3 inhibitors were one of the first molecules to be developed particularly due to their role in the regulation of tumorigenesis (96). One of the molecules that has been developed as a STAT3 inhibitor is of small size as was designed to bind the STAT3 SH2 domain (PY*LKTK), not allowing phosphorylation, an important step for the dimerization and activity at the nucleus (96–98). Several STAT3 inhibitors are currently under investigation in several clinical trials, particularly as immunotherapy targeting oncologic conditions, due to the role of STAT3 as an oncogenic transcription factor (99).

### Autoinflammatory conditions

The definition and recognition of autoinflammatory conditions have been evolving in recent years since this concept was first explored by McDermott et al. in a 1999 publication (100, 101).The authors proposed the use of the acronym TRAPS to define TNF receptor-associated periodic symptoms in conditions with mutations in *TNFR1* and suggested that other mutations in the TNF pathway needed to be explored and could constitute another group of diseases that should be classified as autoinflammatory (100).

In 2017 by *Wekell et al.* that proposed the following definition for auto-inflammatory conditions: “*Autoinflammatory diseases are immunological diseases defined by abnormally increased inflammation, driven by dysregulation of molecules and cells of the innate immune system with a host predisposition as necessary and sufficient criteria, frequently associated with activation of the adaptive immune system and potentially with immune dysfunctions such as susceptibility to infections, autoimmunity or uncontrolled hyperinflammation” *(101).

For this review autoinflammatory conditions are going to be explored below in two groups: Inflammasomopathies and interferonopathies.

#### Inflammasomopathies

**Figure 3:**
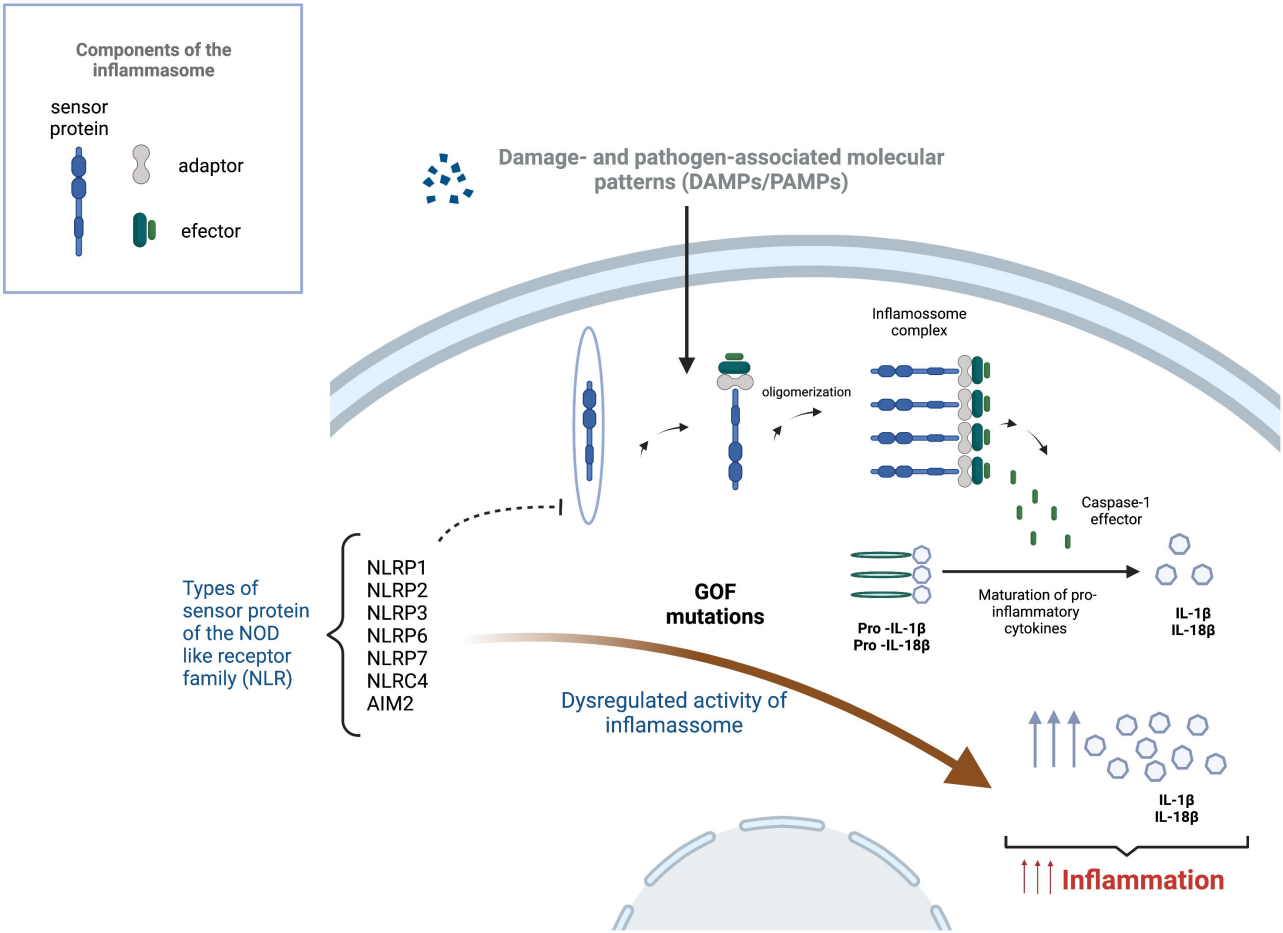
Inflammasome activation and effect of gain of function mutations in the development of inflammasomopathies. Inflammasome activation starts after a trigger, such as damage-associated molecular patterns (DAMPs) or pathogen-associated molecular patterns (PAMPs), is sensed by a sensor protein of the Nucleotide-binding and oligomerization domain (NOD) like receptor family (NLRP1, NLRP2, NLRP3, NLRP6, NLRP7, NLRC4 and AIM2), leading to the oligomerization and formation of multiprotein complex with the ability to cause the cleavage of caspase-1. This enzyme is responsible for the maturation of pro-inflammatory cytokines into their active form. Gain of function mutations (GOF) on the sensor proteins can lead to the dysregulation of the inflammasome causing increased levels of pro-inflammatory cytokines (IL-1 β and IL-18), leading to uncontrolled and damaging auto-inflammatory process.

This group of conditions results from a dysregulated activity of the inflammasomes, a group of intracellular protein complexes that are part of the innate immune system, working as sensors that mediate this type of immune response (**Figure 3**) (102, 103). The activation of the inflammasome is promoted by different endogenous or exogenous stimulus, from both microorganisms, like pathogen-associated molecular patterns (PAMPs) in the case of bacteria, but also signals from fungi, parasites and virus, and from the host, like metabolic or mitochondrial signs, recognised as damage- associated molecular patterns (DAMP) (103, 104). Those signals are identified by inflammasome sensors that initiate the assembly process by recruiting pro-caspase 1 in the canonical pathway and a pro-caspase 11 and 8 in the non-canonical pathway (104, 105).

NLRP3 is one of the best-known sensors inflammasomes and is associated to different monogenic auto-inflammatory diseases.

GOF mutations in *NLRP3 gene* is causative of cryopyrin associated period syndrome (CAPS), a condition that affects several organs and systems including the skin, musculoskeletal and neurologic system with systemic symptoms like fever and fatigue (106). A group of entities initially thought as independent, are currently classified as CAPS, including familial cold autoinflammatory syndrome (FACS), Muckle Wells syndrome (MWS) and neonatal onset multisystem inflammatory disease (NOMID).

The Family Mediterranean fever (FMF) is an inflammasomopathy caused by a mutation *MEFV gene* that encodes the pyrin protein occurring more frequently in eastern Mediterranean populations (102). This autosomal recessive disease is characterised by increased levels of IL-1β leading to recurrent fever episodes, polyserositis and increased levels of acute phase reagents (102).

The mevalonate kinase deficiency (MVK) is caused by autosomal recessive mutation in the *MVK gene*, leading to the accumulation of the mevalonic acid that cause recurrent episodes of fever, enterocolitis, arthritis, cutaneous manifestations and neurological involvement (107, 108). HSCT can be curative, but, due to the rarity of the disease, experience in this cases is scarce and immunomodulator treatment can be considered (108).

Other conditions classified as inflammasomopathies, with increased levels of IL-1β, include the pyogenic arthritis, pyoderma gangrenosum and acne syndrome (PAPA), caused by autosomal dominant mutation in the proline-serine-threonine phosphatase-interacting protein [PSTPIP1, or CD2- binding protein 1 (CD2BP1)] and the NLRC4-related MAS/syndrome of enterocolitis and autoinflammation associated with mutation in NLRC4, the latest also associated to increased levels of IL-18 (102).

The research around the identification of therapeutic targets to treat inflammasomopathies has been targeting the defect in NLRP3. The use of IL-1 signalling inhibitors has been widely explored and used in the current practice (109). Those include canakinumab, an IgG monoclonal antibody that targets IL-1β (109, 110), rilonacept, an IL-1 inhibitor (110) and anakinra, a recombinant from of human IL-1 receptor antagonist (109). More recent studies have been focusing on finding NLRP3 inhibitors, allowing a more targeted approach, precise without a full inhibition of IL. Around ten small molecules have been developed targeting or NLRP3 exclusively or targeting other sensors including NLRP3, NLRC4 and AIM2 (109). Tranilast, an analogue of a tryptophan metabolite, is an approved NLRP3 inhibitor that targets directly the NACHT domain of this protein, impairing the NLRP3 inflammasome assembly (111).

In MVK patients, the use of anti- IL-1 agents induced disease remission with a significative improvement of the histologic and macroscopic findings in the gastrointestinal tract and other autoinflammatory disease manifestations (108, 112). In this disease, the decrease of isoprenoid end-products due to the accumulation of mevalonic acid leads to increased levels of IL-1β that contribute for a pro-inflammatory state, explaining the mechanism of action of anti- IL-1 agents (108).

#### Interferonopathies

Interferons can be classified in three classes: the type I family including IFN-α variants and IFN-β, type II family with IFN-γ and type III family (IFN-λ) (105, 113). Type I IFN can be induced by microbial nucleic acids recognised by cytosolic RNA helicases and cytoplasmatic DNA receptors (cyclic GMP-AMP synthase) (114). The cGMP-AMP activate the stimulator of interferon genes (STING) that after recruiting TANK-binding kinase 1 (TBK1), activating IRF7 and IRF3 regulatory factors that in the nucleus lead to the transcription of IFN-α and IFN-β.

In the canonical type I interferon signaling pathway, the type I IFN bind to the IFN-α receptor (IFNAR), activating the receptor-associated protein tyrosine kinases, Janus kinase 1 (JAK1) and tyrosine kinase 2 (TYK2), resulting in the phosphorylation of STAT 1 and 2. After migrating to the nucleus they form the IFN-stimulated gene factor 3 (ISGF3) that lead to the expression of interferon stimulated genes (ISGs) (114, 115) The relevance of a strict regulation of this pathway is to avoid uncontrolled IFN responses, that contribute to excessive inflammation.

Aicardi-Goutières syndrome (AGS) was one of the first conditions to be described in this group of interferonopathies and is characterized by a progressive encephalopathy in the first year of life and a range of extra-neurologic manifestations including vasculopathy, interstitial lung disease, hepatic disease and psoriasis (116).

The proteasome-associated auto-inflammatory syndromes (PRAAS) are a group of conditions that are the result of a LOF mutations in genes that encode proteasome subunits (PSMB7, 8 and 9, PSMA3) and other proteasome chaperone factors (POMP and PSMG2) (114). The CANDLE syndrome (chronic atypical neutrophilic dermatosis with lipodystrophy and elevated temperature) first described in 2010 (117), is a well recognise PRAAS caused by an impairment to remove waste proteins from the cell, leading to an underlying inflammation augmented by basic triggers, like stress, physical activity or a viral infections (118).

Another rare condition is the spondyloenchondrodysplasia with immune dysregulation (SPENCD), a skeletal dysplasia, neurological disease and immune dysregulation, caused by a autosomal recessive mutation in the tartrate-resistant acid phosphatase (TRAP) gene (*ACP5 gene*) (119).

Other conditions in this group, with different phenotypes include: monogenic systemic lupus erythematous (*TREX1, SAMHD1 genes)*, ISG15 deficiency (*ISG15 gene*), Singleton-Merten syndrome (*IFIH1 gene*), trichohepatoenteric syndrome (*SKIV2L gene*), X-linked reticulate pigmentary disorder (*POLA1 gene*), STING- associated vasculopathy of infancy (*TMEM173 and STING genes*) and COPA *(COPA gene)* (105, 114).

The application of precision medicine in interferonopathies have the aim to control the outcomes caused by the dysregulation of this pathway by limiting the production, increase removal or degradation of nucleic acids or by blocking or reducing the pathway signaling (115, 120). With several clinical trials ongoing targeting different molecules, the JAK1 inhibitors have been used in clinical setting with better outcomes in skin and systemic manifestations when compared with neurologic disease (115, 120, 121).

### Hemophagocytic lymphohistiocytosis

HLH is a life-threatening condition that results due to uncontrolled activation of macrophages, NK cells and cytotoxic T lymphocytes (122). After the first diagnostic guidelines in 1991, in 2004 the Histiocyte Society defined a total of eight criteria as part of the diagnosis for HLH, including the most common clinical manifestations like fever, splenomegaly and laboratory alterations: cytopenias of at least two lineages, hypertriglyceridemia or hypofibrinogenemia, hemophagocytosis in primary and secondary lymphoid organs, low or absent NK cell activity, hyperferritinemia and increased levels of IL-2 (123). For the disease to be diagnosed the patient needs to fulfil five of the eight criteria described or have a confirmed genetic mutation (123).

With the concept that this condition can be primary or secondary to a specific immunologic trigger, in 2018, the same Society determined that this condition could be organised in three categories: 1) primary “familial” HLH, for inherited conditions identified because of the presence of the same condition in a family member or in case of the identification of a causative mutation; 2) Macrophage activation syndrome (MAS), when the inflammation process is triggered by an auto-immune condition, 3) cases of HLH secondary to a specific underlying medical condition, including infection, malignancy, primary immunodeficiency or a metabolic disease (124).

Several genetic defects identified in primary HLH were associated to a specific immune function: cytotoxic granule content (perforin 1 - *PRF1 gene*), cytotoxic exocytosis pathway (*UNC13D, STX11, STXBP2, RAB27A, LYST gene)*, cytotoxic T-cell signaling (*SH2D1A gene*), inflammasome regulation and activation (*BIRC4, NLRC4 gene*) (125). The relevance of identifying a genetic defect in HLA patients is the possibility to propose a curative treatment, like HSCT (124). Immuno and chemotherapy schemes have been successfully used to induce remission (123, 126). One of the challenges when using a conditioning treatment that would control effectively the hyperinflammation state in HLH patients, is that the heterogenicity of causes can lead to different outcomes (124). Survival rates without HSCT are low, but even with this intervention long-term survival rates for patients with XIAP deficiency do not reach 50% (127), similar to XLP patients with active HLH at the time of the transplant (128).

By identifying the underlying pathway that is affected, it is possible to use immunomodulatory treatments, targeting the defect, like the use of JAK inhibitor in case of a STAT3 GOF mutation, the potential use of recombinant human Il-18 binding protein (rhIL-18BP) in NLRC4 mutations or the use of monoclonal antibody against IFN-γ in MAS-HLA (124, 129, 130), reducing the severe sides effects associated to aggressive chemotherapy treatments and potentially with a favourable outcome. For XIAP deficiency patients, a phase 3 randomized clinical trial is ongoing (ClinicalTrials.gov Identifier NCT03113760) using rhIL-18BP, a protein that will regulate the elevated IL-18 cytokine levels that these patients manifest recurrently (131). A clinical case report of a patient with XIAP not responsive to several medications, entered in remission after administration of rhIL-18BP, with free IL-18 levels below 10.00 pg/mL (131).

The option of gene therapy in HLH patients is very challenging in comparison with other PID, since the patient T cells are hyperactivated and the mechanisms for correction of the gene defect can be changed by this phenomenon. This characteristic can lead to the need of using remission methods similar to HSCT, before any cell can be collected and genetically treated, taking away one of the main advantages of gene therapy, of non or less aggressive conditioning treatment. Independently of the challenges, gene therapy has been evaluated as a treatment option in these patients. An animal model (mice) was developed as a proof of concept that it was possible to correct *PRF1 gene* mutations in CD8 murine T cells with resolution of the immune dysregulation manifestations (132, 133). The same authors tested this approach in human T cells using a lentivirus (132). Another mutation causative of familial hemophagocytic lymphohistiocytosis (type 3), *UNC13D gene* mutation, was also evaluated as a potential target for gene therapy in a mice model using a self-inactivating lentiviral vector, showing an ability to correct the gene mutation and the cell cytotoxic activity (134). Clinical trials in individuals with X-link lymphoproliferative (XLP) syndrome, causative of defects in the SAP protein are in set up (128), after successful investigations in a murine model, using a lentiviral vector. In this model the gene correction restored the cytotoxic activity of NK cells and of the humoral activity of T cell dependent responses (135).

## Target therapies in precision medicine

A general perspective and evolution of immunomodulators drugs, is presented below.

### Biologics and small molecules

The use of biologic therapeutics and small molecules that target the immune pathway or the molecular defect is one of the most relevant and fast evolving fields in PID. The identification of specific gene variants allows to identify what precise molecules need to be replaced, what products need to be eliminated or what molecules should be inhibited. The more these treatments are precise in the target, less the number of expected side effects and higher is the probability to control disease manifestations. In certain PID, particularly with high severity, the use of HSCT is still the most adequate and potentially the only curative treatment (136). Even in these cases there is a role for these therapeutic agents, since by controlling disease manifestations it can work as a bridge to HSCT (136).

**Table 1** summarises the most relevant therapeutic groups that work as targeted treatments for PID.

**Table 1:** Targeted treatments used in primary immunodeficiency (PID).

Drug group	Name of the drug	Type of molecular structure	Target	Mechanism of action	Indications in PID (including off-label)	Approvals and dosing
JAK Inhibitors (130, 137–140)	Ruxolitinib	Small molecule	JAK1 and JAK2	Competitively binds to the adenosine triphosphate-binding site of JAK and inhibits the enzyme activity of JAK leading to suppression of cytokine signal transduction and cytokine action	STAT3-GOF STAT1-GOF CANDLE synd. HLH Interferonopathies	FDA* and EMA*
	Tofacitinib	Small molecule	JAK1 and JAK3	Competitively binds to the adenosine triphosphate-binding site of JAK and inhibits the enzyme activity of JAK leading to suppression of cytokine signal transduction and cytokine action	STAT3-GOF STAT1-GOF CANDLE	FDA* and EMA*
	Baricitinib	Small molecule	JAK1 and JAK2	Competitively binds to the adenosine triphosphate-binding site of JAK and inhibits the enzyme activity of JAK leading to suppression of cytokine signal transduction and cytokine action	STAT1-GOF CANDLE synd.	FDA* and EMA*
TNF-α Inhibitors (130, 140, 141)	Infliximab	Chimeric (human/murine) monoclonal IgG1 antibody	TNF-α	Binds to both soluble subunit and the membrane-bound precursor of TNF-α causing a disruption of the interaction of TNF-α with its receptors	CANDLE synd. POMP deficiency PAPA synd. Blau synd. CVID STAT3 GOF	FDA*♦ and EMA*♦ ♦(*IBD phenotypes – 5 mg/kg every 8 weeks after induction)*
	Adalimumab	Recombinant human monoclonal IgG1 antibody	TNF-α	Binds to both soluble subunit and the membrane-bound precursor of TNF-α causing a disruption of the interaction of TNF-α with its receptors	CANDLE synd. POMP deficiency PAPA synd. Blau synd. CVID	FDA*♦ and EMA*♦ ♦(*IBD phenotypes-* *<*40kg: 20 mg/week ♦40 mg: 40 mg/week or 80 mg every other week after induction)
	Etanercept	Dimeric fusion protein	TNF-α	By competitive inhibition prevents TNF to bind to cell surface, inhibiting the pro-inflammatory activity of TNF-α	CANDLE synd. POMP synd. PAPA synd. Blau synd. CVID	FDA* and EMA*
IFN-γ inhibitor (130, 140, 142)	Emapalumab	Human monoclonal IgG1 antibody	IFN-γ	Binds to free and receptor-bound interferon-γ, inhibiting receptor dimerization and transduction of interferon-γ signaling, leading to an inhibition of its biologic activity	HLH	FDA♦ ♦ *(HLH – 1 mg/kg twice a week)*
Interleukin antagonist or interleukin binding drugs (110, 130, 140, 143–146)	Anakinra	Nonglycosylated, recombinant, human interleukin-1 receptor antagonist	IL-1R	Binds competitively to the Interleukin-1 type I receptor (IL-1RI), inhibiting the action of elevated levels IL-1, a proinflammatory cytokine	CASP	FDA*♦ and EMA*♦ ♦ *(CASP- 1 -2 mg/kg/day)*
	Canakinumab	Human IgGκ monoclonal antibody	IL- 1β	Binds to human IL-1β and neutralizes its inflammatory activity by blocking its interaction with IL-1 receptors	CAPS TRAPS MKD	FDA*♦ and EMA*♦ ♦*(CASP - >* 40* kg*:150 mg 15-40 kg:2mg/kg 7.5-15 kg: 4 mg/kg Every 8 weeks) ♦ *(TRAPS, MKD-* *- >* 40* kg*:150 mg 7.5-40 kg: 2 mg/kg Every 4 weeks)
	Rilonacept	Dimeric fusion protein	IL- 1β	Blocks IL-1β signaling by acting as a soluble decoy receptor, preventing interaction of IL-1β with cell surface receptors	CAPS	FDA♦ ♦ *(CASP* ♦ 18 years:320 mg at first dose; 160 mg following doses once a week 12-17 years: 4.4 mg/kg (maximum 320 mg) in the first dose, 2.2 mg/kg following doses (maximum 160 mg Once a week)
	Tocilizumab	Recombinant humanized monoclonal antibody	IL-6R	Inhibits the binding of interleukin-6 (IL-6) to its receptor (IL-6R), preventing IL-6 signal transduction to inflammatory mediators that stimulate B and T cells.	STAT3-GOF	FDA* and EMA*
	Tadekinig-alfa	Recombinant human IL-18 binding glycoprotein	IL-18	Binds to free IL-18, a proinflammatory cytokine of the IL-1 family that is produced by various cell types, including monocytes/macrophages, preventing its binding to the receptor and consequently the IL-18 biologic activity	NLCR4-GOF	Granted orphan designation by FDA and EMA
	Ustekinumab	Human immunoglobulin (Ig) G1 kappa monoclonal antibody	IL-12 and IL-23 (p40 subunit)	Binds to the p40 subunit common to IL-12 and IL-23 and prevents their interaction with the IL-12 receptor β1 subunit of the IL-12 and IL-23 receptor complexes, neutralizing human IL-12- and IL-23-mediated cell signaling, activation and cytokine production.	LAD CGD-colitis	FDA*♦ and EMA♦ ♦(*IBD phenotypes* ≤55 kg:260 mg 55-85Kg: 390 mg >85 mg: 520 mg)
Modulator of CD80/86 (130, 136, 140, 147)	Abatacept	Fusion protein (CTLA4 -Ig)	CD80/CD86	Binds to CD80/CD86 receptors, preventing the interaction with the CD28 receptor, blocking the signal for immune activation of T cells and antigen-presenting cell	CTLA-4 haploinsufficiency LRBA deficiency	FDA* and EMA*
	Balatacept	Fusion protein (CTLA4 -Ig) 2^nd^ generation	CD80/CD86	Binds to CD80/CD86 receptors, preventing the interaction with the CD28 receptor, blocking the signal for immune activation of T cells and antigen-presenting cell	CTLA-4 haploinsufficiency LRBA deficiency	FDA* and EMA*
B cell directed therapies (140, 148, 149)	Belimumab	Human recombinant IgG1λ monoclonal antibody	B cell	Inhibits B-lymphocyte stimulator (BLyS), and indirectly inhibits B cell survival (autoreactive B-cell apoptosis)	Autoimmune cytopenias	FDA* and EMA*
	Bortezomib	Dipeptide boronic acid derivative	Plasma cells	Inhibits the 26S proteasome, a protein complex that degrades ubiquitinated proteins in the ubiquitin-proteasome pathway, leading to cell cycle arrest and apoptosis	Autoimmune cytopenias	FDA* and EMA*
	Epratuzumab	Anti-CD22 humanized monoclonal antibody derived from the murine IG2a monoclonal antibody	CD22	Contributes to an alteration of the B cell receptor signaling complex, reducing signaling and activation of B cells	Autoimmune cytopenias	
	Ibrutinib	Small molecule	Burton’s tyrosine kinase	Inhibits Burton’s tyrosine kinase leading to perturbation of B-cell development due to the role of this target in the B-cell receptor signaling pathway	Autoimmune cytopenias	FDA* and EMA*
	Rituximab	Chimeric murine/human monoclonal antibody against the CD20	CD20	After binding to CD20, rituximab mediates B-cell lysis (or breakdown). The possible mechanisms of cell lysis include complement dependent cytotoxicity (CDC) and antibody dependent cell-mediated cytotoxicity (ADCC	Autoimmune cytopenias Granulomatous lymphocytic interstitial lung disease Granulomatous disease	FDA* and EMA*
Mammalian target of rapamycin (mTOR) inhibitor (13, 150, 151)	Sirolimus	Small molecule (macrolide antibiotic)	mTOR	inhibition of the mTOR, which is a serine/threonine-specific protein kinase that regulates cell growth, proliferation, and survival.	NLCR4-GOF POMP deficiency CTLA-4 haploinsufficiency APDS	FDA* and EMA*

APDS, Activated PI3K Delta Syndrome; CANDLE, Chronic Atypical Neutrophilic Dermatosis with Lipodystrophy and Elevated temperature; CAPS, cryopyrin-associated periodic syndrome; CGD, chronic granulomatous disease; CTLA-4, cytotoxic T lymphocyte antigen-4; CVID, common variable immunodeficiency; EMA, European Medicines Agency; FDA, Food and Drug administration; GOF, Gain of function; HLH, Hemophagocytic lymphohistiocytosis; JAK, Janus kinase; LAD, leukocyte adhesion deficiency; MKD, mevalonate kinase deficiency; mTOR, mammalian target of rapamycin; PAPA, pyogenic arthritis, pyoderma gangrenosum, and acne; POMP, Proteasome maturation protein; TRAPS, tumor necrosis factor receptor associated periodic syndrome.

*Approved for other generic conditions not specified in this table.

♦ Conditions that can be considered as part of the PID described.

#### JAK Inhibitors

The JAK-STAT pathway is responsible for the transduction of signals of different molecules, from cytokines to hormones, modulating immune responses (130, 137). In PID, different mutations can affect the JAK-STAT pathway, leading to immune dysregulation and autoimmunity manifestations. JAK family is composed by four tyrosine protein kinases (JAK1, JAK2, JAK3 and TYK2) and the STAT protein family is composed by STAT1, STAT2, STAT3, STAT4, STAT5a, STAT5b andSTAT6, showing the complexity and diversity of this pathway (58, 152) The cytokines and other molecules bind to JAK type I and II receptors activating the phosphorylation of tyrosine residues and creating a biding site for STAT proteins to attach and be activated by phosphorylation. This process allows STAT protein to be translocated to the nucleus and to bind to the DNA, regulating the gene expression of several genes involved in immune responses (58, 137, 152).

**Figure 4:**
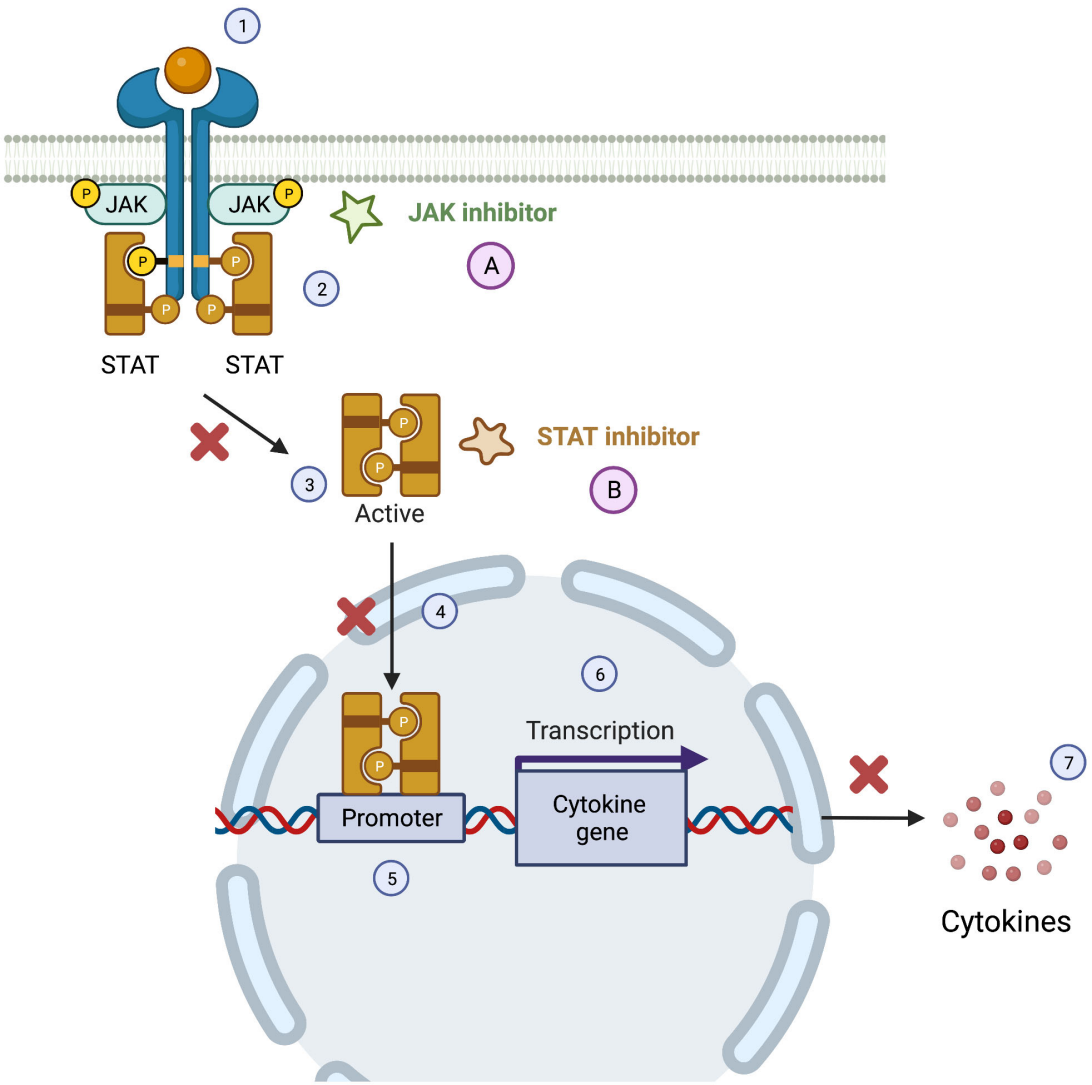
JAK-STAT pathway and the therapeutics targets of JAK and STAT inhibitors. The Janus Kinase/signal transducer and activator of transcription pathway (JAK/STAT) can be activated by cytokines (1), leading to a phosphorylation of JAK, that in the active form allows the docking of STATs and consequent phosphorylation of this molecules (2). After the phosphorylation of STATs they dissociate for the JAK receptor (3) and the STAT active form translocate to the nucleus (4) targeting the gene promoter (5) causing the transcription of cytokine gene (6) and consequently the production of cytokine (7). JAK inhibitors **(A)** and STAT inhibitors **(B)** can interfere with this pathway leading to a reduced production of pro-inflammatory cytokine.

Specifically targeting the JAK-kinase pathway, two groups of molecules need to be considered (**Figure 4**): JAK and STAT inhibitors, the latest still in early clinical trials phases or pre-marketing approval for PID (58).

JAK inhibitors are small molecules with a dose-dependent selectivity to specific JAK members (table 1), that interfere with JAK-STAT signaling pathway reducing pro-inflammatory cytokines and other subtracts (58, 153). First generation JAK inhibitors (tofacitinib, baricitinib, and ruxolitinib) were the first to be developed and tested, but have a lower specificity when compared with the newer second-generation molecules (JAK-1 inhibitors: filgotinib and upadacitinib; JAK-2 inhibitors: fedratinib) (58, 137).

Tofacitinib was the first JAK inhibitor to be used in humans. It targets in first place JAK1 and JAK3 and is the molecule that was better studied in terms of safety (58, 154). Baricitini and ruxolitinib are both selective to JAK 1 and 2 (58).

Several other JAK-inhibitors are also under evaluation: abrocitinib (selective for JAK1), itacitinib (selective for JAK1), pacritinib (selective for JAK2), gandotinib (selective for JAK2), decernotinib (selective for JAK3), peficitinib (selective for JAK3), momelotinib (selective for JAK1 and JAK2), gusacitinib (selective for JAK1, JAK2, JAK3 and TYK2), delgocitinib (selective for JAK1, JAK2, JAK3 and TYK2) and cerdulatinib (selective for JAK1, JAK2 and TYK2) (58, 137).

For PID, JAK inhibitors have been used in a range of conditions. A Jak1/JAK2 inhibitor, baricitinib with a mean dose 8.5 mg/day, was used as part of a clinical trial in patients with CANDLE syndrome (155). After six months of follow-up the authors reported an improvement in the auto-inflammatory diary score, a decrease in prednisolone dosage and an improvement of the haematologic series (155). This drug was used recently in a patient with a CANDLE syndrome (PRAAS) caused by a compound heterozygous mutation in the *PSMB8* gene with significant clinical improvement, at a maintenance dosage of 8mg/day (138). For other conditions, JAK-inhibitors have been used in a small number of patients with good outcomes, including STING-associated vasculopathy with onset in infancy, Aicardi-Goutières syndrome (AGS) and mutations interfering with IFN signaling (137).

In patients with STAT-1 GOF a retrospective study including 10 children treated with JAK inhibitors (nine with ruxolitinib and one with baricitinib) reported an improvement of the immune deficiency and dysregulation activity score for the patients treated with ruxolitinib with variations at the time of remission depending on the disease manifestations (156). The doses for ruxolitinib approved by FDA vary depending on the condition and for children with PID are not determined (156, 157). In a review by Deya-Martinez et al. the median starting dose of ruxolitinib was 0.3 mg/kg/day, with a maximum of 0.6 mg/kg/day and the median dose of baricitinib of 2 mg/day with a median maximum dose of 4 mg/day (156).

The treatment of STAT3 GOF mutations is challenging and still with no consensus (158). A patient with a life-threatening condition, type 1 diabetes and interstitial lung disease under mechanical ventilation was treated with an anti- IL-6 receptor, tocilizumab, combined with a JAK inhibitor ruxolitinib at a total dose of 2.2 mg/kg/day, with a regime of three times a day, instead of the twice a day recommended for other diseases (158). Another patient with type 1 diabetes, lipodystrophy and lymphoproliferative disorder was treated with ruxolitinib at a dosage of 15 mg/m2/day to a maximum of 40 mg/m2/day, with a reduction of the insulin needs and of auto inflammatory/lymphoproliferative manifestations (93).

In case of HLH, studies in a mice model showed a favourable response to ruxolitinib, with an increase in the mice survival rates, tissue repair and improvement of inflammatory responses (159). After the use in the animal model, ruxolitinib has been used in individuals with primary or secondary HLH, with a dosage that varied from 2.5 mg to 20 mg twice a day (139). In the paediatric population the doses varied from 0.3 mg/kg/day or 2.5 mg in children with less than 10 kg, 5 mg between 10-25 kg and 10 mg if weight >25 kg (139).

The reported side effects include an increased susceptibility to infectious diseases, particularly tuberculosis and reactivation of herpes zoster and an increased risk of development of non-melanoma skin cancer, as well as a theoretical increased risk of other malignancies, cardiovascular disease and cytopenias (152, 153). In an 8.5 years follow-up study of individuals treated with tofacitinib, the adverse events reported were stable over time, showing the long-term safety of the use of this treatment, although there is still a need to expand the knowledge in this area (153, 154). It is considered that the side effects associated to JAK inhibitors are similar for all the different molecules.

#### Anti-TNF-α agents

TFN superfamily are a group of cytokines, including TNF-α that can present as transmembrane or a soluble form in the plasma, both able to bind to TNFR1 and TNFR2 (141). TNF-α is one of the most relevant proinflammatory cytokine and is able to activate immune cells from both innate and adaptative immune system, activate proinflammatory cytokines and inhibit regulatory T cells (141).

Anti-TNF-α agents (**Table 1**) can control immune dysregulation in conditions with autoinflammation like PIRDs. Anti-TNF-α agents have a particular role in the treatment of interferonopathies and autoimmune diseases (160). The three most common used anti-TNF-α agents are: infliximab (chimeric mouse/human) administered previously in patients with CVID, ataxia-telangiectasia, leucocyte adhesion deficiency, STAT3 GOF mutations, NEMO deficiency; adalimumab (human) in patients with LBRA mutation, CVID and STAT3 GOF mutations and etanercept in patients with CVID (141).

Infliximab was recognised as a therapeutic agent for patients with CVID with granulomatous disease, a condition that can increase morbidity and mortality in these patients (161). In a case series of PID patients with granulomatous disease, classified as CVID, infliximab 5 mg/kg with two weeks intervals for induction (0, 2 weeks) and every 4 weeks afterwards was used for a mean period of 9.4 months. A significant improvement of the symptoms and radiologic findings were identified in these patients (161). The same principle is applicable to other PID with associated granulomatous disease, like ataxia-telangiectasia (141). The indications for infliximab can be extended for other PID with gastrointestinal manifestations in the spectrum of IBD. In these patients the outcomes are variable, and the use of an anti-TNF drug was commonly associated with other medications (162). Infliximab use was reported in a patient with IPEX syndrome (immunodysregulation, polyendocrinopathy enteropathy X-linked) in the dosage of 5 mg/kg (0,2,6 and then every 8 weeks) with gastrointestinal manifestations and spondylarthritis, with significant improvement of the articular and gastrointestinal symptoms and with an increase of the levels of FOXP3+ CD4+ Tregs (163). Adalimumab as similar indications as infliximab with the advantage in the type of administration (subcutaneous) in comparison to infliximab (intravenous) with doses that can vary depending on the patient weight and disease (141). Etanercept is also administrated in a subcutaneous route, typically twice a week in a dosage of 25 mg or 0.4mg/kg or the double of the dosage when administered once weekly (164, 165). The use of etanercept is described in patients with CVID and granulomatous disease (164)

The most common side effects associated to the use of anti-TNF-α agents are the higher susceptibility to disseminated infections caused by bacteria and fungi and reactivation of latent tuberculosis (141).

#### IFN- γ inhibitors

IFN- γ is a cytokine with a role in immune responses against bacteria, inflammation, auto-immunity, and antigen processing. It is produced by T cells and NK cells and is able to induce JAK-STAT1 pathway and the Toll-like receptor (TLR) pathway that activated GTPase, the latest with a role in the elimination of intracellular pathogens *via* Il-12/IFN- γ axis (166).

Emapalumab is a human monoclonal IgG1 antibody that binds to free and receptor-bound interferon-γ, inhibiting receptor dimerization and transduction of interferon-γ signaling, leading to a blocking of this cytokine activity (142, 166).

Due to role of IFN- γ in HLH, responsible for causing hyperinflammation, inhibition of high levels of IFN- γ can work as a control strategy for this disease. This drug was evaluated in a clinical trial, including 34 patients with primary hemophagocytic lymphohistiocytosis that received emapalumab at an initial dosage of 1mg/kg every 3 days and after adjustments increased the dosages of 3, 6 until a maximum of 10 mg/kg (142). In this study a decrease in the serum levels of CXCL9 was identified and it was possible to decrease progressively steroid dosages. It was considered that the adverse events profile was similar to other drugs. Although the number of patients was low, not allowing to make any definitive conclusions of the role of emapalumab in HLH, apparently the outcomes were better in the patients submitted to HSCT with emapalumab when compared with the patients that did not receive emapalumab (142). A pharmacometrics model was also developed to determine the correct dosages of emapalumab in HLH and consequent outcomes (167). The authors found high levels of intra and inter- individual variation of IFN- γ levels and that the reduction of those levels could lead to general improvement. The doses studied were the same as previous reported, with an initial dose of 1 mg/kg, with progressive increase for 3, 6 or 10 mg/kg every 3 days based on disease activity, concluding the need for dose adaptation (167).

#### Interleukin antagonists or interleukin binding drugs

Interleukin antagonists or interleukin binding drugs target specific cytokines that are involved in the autoinflammatory process of different PID as described in **Table 1**. IL-1 antagonists are diverse and one of the most studied anti-IL drugs. IL-1 is a potent proinflammatory cytokine that can be classified as IL-1-α or IL-1β. They bind to the same receptor, stimulating an intracellular kinase dependent signaling process that contribute for cell activation processes part of innate immunity and to the activation of B cells *via* NFkB pathway (168). Currently different pharmacological agents can be used to inhibit IL-1 targeting different components, the IL-1 receptor (anakinra) (130) or the IL-1 molecules by the use of a monoclonal antibody (canakinumab) anti-IL-1 or a fusion protein that inhibits the binding of IL-1 to receptor (rilonacept) (110). IL-1 inhibitors are used in treatment of auto-inflammatory diseases, like monogenic periodic fever syndromes including CAPS and TRAPS (169). In a review of the current available literature, including clinical trials and registry-based trials, the authors concluded that the use of these drugs allowed the control of the disease activity and inflammatory markers, including the possibility of tapering other associated medications like steroids (169). In the evaluation of the published trials, the dosages varied depending on the disease (CASPS: Canakinumab 2 mg/kg or 150 mg/dose if >40 Kg; anakinra 0.5-2.5 mg/kg/dia, or 100 mg if >40 Kg; TRAPS: Canakinumab 150 mg/dose or 2 mg/kg; anakinra 1.5 mg/kg/dose) and no superiority of anakinra when compared with canakinumab was identified (169).

Tocilizumab is a recombinant humanized monoclonal antibody that inhibits the binding of interleukin-6 (IL-6) to its receptor (IL-6R), preventing IL-6 signal transduction to inflammatory mediators that stimulate B and T cells (143). This drug was initially studied for rheumatoid arthritis at a dosage of 4-8 mg/kg with infusions every 4 weeks (170). Currently is used in patients with STAT-3 GOF (171). STAT-3 is part of a pathway that stimulates the production of IL-6 and GOF mutations, has previously described, are associated to auto-inflammatory manifestations, hypogammaglobulinemia and other conditions like type I diabetes. At a dosage of 8 mg/kg every two weeks, after 2 months treatment with toculizumab, a remission of the pancytopenia was documented, as well as the need for insulinotherapy (171). Tocilizumab has also been administrated in patients with RAG deficiency, although the full associated mechanism and long-term effects are not known (172).

Tadekinig-alfa is a recombinant human IL-18 binding glycoprotein, that binds to free IL-18, a proinflammatory cytokine of the IL-1 family that is produced by various cell types, including monocytes/macrophages, preventing its binding to the receptor and consequently the IL-18 biologic activity. This medication was used in GOF mutations of an inflammasome protein, NLRC4, at a dosage of 2 mg/kg every 48h, with an improvement of the laboratory markers, gastrointestinal manifestations, and with progressive reduction of the other immunosuppressive medication (173).

Ustekinumab is a human immunoglobulin (Ig) G1 kappa monoclonal antibody that binds to the p40 subunit common to IL-12 and IL-23 and prevents their interaction with the IL-12 receptor β1 subunit of the IL-12 and IL-23 receptor complexes, neutralizing human IL-12- and IL-23-mediated cell signaling, activation and cytokine production (174). After animal studies in a mice model, ustekinumab was already used in humans with the aim to block the activity of IL-12 and Il-23 and consequently downstream IL-17, with symptoms remission (174).

#### Modulator of CD80/86

Abatacept and balatacept (2^nd^ generation) are fusion proteins (CTLA4 -Ig) that bind to CD80/CD86 receptors, preventing the interaction with the CD28 receptor and blocking the signal for immune activation of T cells and APCs.

Abatacept can be used as a targeted immunomodulatory treatment for LBRA defects and CTLA4 haploinsufficiency, by competing with CD28 for binding of CD80 and CD87, inhibiting T cell activation, with a dosage that vary by weight (recommended 10 mg/kg every 4 weeks for paediatric patients with less than 75 kg) (69, 78). It was shown that the use of CTLA4-Ig in patients with autoimmune enteropathy was associated to an improvement of the gastrointestinal symptoms, like diarrhoea and inflammatory manifestations in general (175–177).

#### B cell targeted therapies

B cell targeted therapies are an important advance in the treatment of auto-immune diseases (178). Different types of monoclonal antibodies targeting B cells have been developed, inhibiting of B cell function, survival and activation (150, 178). Rituximab is a chimeric murine/human monoclonal antibody against the CD20 that after binding to CD20, rituximab mediates B-cell lysis or breakdown (140). The possible mechanisms of cell lysis include complement dependent cytotoxicity (CDC) and antibody dependent cell-mediated cytotoxicity (ADCC) (148). In PID rituximab has been used in patients with auto immune cytopenias. In a review of 33 patients with CVID and immune thrombocytopenia (ITP) and/or autoimmune haemolytic anaemia (AHA) treated with rituximab, a dosage of 375 mg/m2 was used once a week for 4 weeks in the majority of the patients (179). The use of this treatment contributed to remission in 85% of the cases with a long-term response in 50% of the cases. The most common adverse event was severe infection in 24% (179).

Other monoclonal antibodies have been under evaluation and used as an alternative for auto-immune cytopenias including: belimumab a human recombinant IgG1λ monoclonal antibody that inhibits B-lymphocyte stimulator (BLyS), and indirectly inhibits B cell survival (autoreactive B-cell apoptosis) (180); bortezomib a dipeptide boronic acid derivative that inhibits the 26S proteasome, a protein complex that degrades ubiquitinated proteins in the ubiquitin-proteasome pathway, leading to cell cycle arrest and apoptosis (181); epratuzumab an anti-CD22 humanized monoclonal antibody derived from the murine IG2a monoclonal antibody that contributes to an alteration of the B cell receptor signaling complex, reducing signaling and activation of B cells (149) and ibrutinib is a small molecule that inhibits burton’s tyrosine kinase leading to perturbation of B-cell development due to the role of this target in the B-cell receptor signaling pathway (182). Daratumumab is a monoclonal antibody that targets CD38 receptors, and currently approved by EMA and FDA for multiple myeloma and light chain amyloidosis. CD38 is expressed in several cells, including hematopoietic cells and has been used in patients with autoimmune haemolytic anaemia, with low side effects (183), although more data is needed to determine it's role in autoimmune diseases.

#### Mammalian target of rapamycin inhibitors

Mammalian target of rapamycin (mTOR) inhibitors target mTOR signaling pathway that regulates immune metabolism and function with a particular role in the T-helper (Th) 1, Th2, Th17 and Treg cell differentiation, CD8+ T cell trafficking and development of CD8+ T memory cells (184). mTOR are part of the phosphatidylinositol-3-kinase-related kinases (PIKKs) family, sharing similar sequencing to phosphatidylinositol-3-kinase (PI3K) (185). Rapamycin (sirolimus) is a natural product and the first mTOR that was developed. Other rapamycin analogues were developed later (temsirolimus, everolimus and ridafarolimus) showing a better bioavailability (185). mTOR inhibitors have a particular role in T cell mediated diseases (151).

Although the significant advances in the discovery of biologic agents and new molecules that are already improving patient life-quality and disease outcomes, data regarding the dosages adapted to the disease and patient characteristics, timing of drug initiation, duration of use and long-term side effects are still scarce, and more research is needed in PID patients.

## Conclusion/final remarks

The wider use of NGS techniques allowed to identify an increased number of pathogenic genetic variants in PID patients (13, 186, 187). This led to a better understanding of the immune mechanisms affected and the underlying genetic causes. This step is fundamental to determine the use of targeted treatments to each specific defect, like small molecules or other biologic compounds or to define with more precision and with better outcomes the candidates to curative treatment with HSCT or even with the use of gene therapy.

It is likely that the future in PID is the use of a precision medicine approach, increasing the use of genetic diagnostic methods and with a full characterization of the genome, like whole genome sequencing and to increase research investment in molecular targeted therapies for orphan/rare disorders.

## Author contributions

MVP analysed evidence from literature and wrote the manuscript. JN analysed evidence from literature and critically reviewed the final draft. All authors contributed to the article and approved the submitted version.
